# Puumala Orthohantavirus Reassortant Genome Variants Likely Emerging in the Watershed Forests

**DOI:** 10.3390/ijms24021018

**Published:** 2023-01-05

**Authors:** Emmanuel Kabwe, Anton F. Shamsutdinov, Setora Suleimanova, Ekaterina V. Martynova, Ruzilya K. Ismagilova, Venera G. Shakirova, Tatiana A. Savitskaya, Guzel S. Isaeva, Albert A. Rizvanov, Svetlana F. Khaiboullina, Sergey P. Morzunov, Yuriy N. Davidyuk

**Affiliations:** 1 OpenLab “Gene and Cell Technologies”, Institute of Fundamental Medicine and Biology, Kazan Federal University, 420008 Kazan, Russia; 2 Kazan Research Institute of Epidemiology and Microbiology, 420012 Kazan, Russia; 3 OpenLab “Omics Technology”, Institute of Fundamental Medicine and Biology, Kazan Federal University, 420008 Kazan, Russia; 4 Medical Academy of the Ministry of Health of the Russian Federation, 420012 Kazan, Russia; 5 Department of Pathology, University of Nevada, Reno, NV 89557, USA

**Keywords:** *Puumala orthohantavirus*, hemorrhagic fever with renal syndrome (HFRS), genome variants, reassortment

## Abstract

Hemorrhagic fever with renal syndrome (HFRS) remains a prevalent zoonosis in the Republic of Tatarstan (RT), Russian Federation. *Puumala orthohantavirus* (PUUV), carried by bank voles (*Myodes glareolus*), is the principal zoonotic pathogen of HFRS in the RT. In this study, we sought to demonstrate the similarity of the PUUV genetic sequences detected in HFRS case patients and bank vole samples previously collected in some areas of the RT. Furthermore, we intended to identify the reassortant PUUV genomes and locate a potential site for their emergence. During 2019 outbreaks, the PUUV genome sequences of the S and M segments from 42 HFRS cases were analysed and compared with the corresponding sequences from bank voles previously trapped in the RT. Most of the PUUV strains from HFRS patients turned out to be closely related to those isolated from bank voles captured near the site of the human infection. We also found possible reassortant PUUV genomes in five patients while they were absent in bank voles. The location of the corresponding HFRS infection sites suggests that reassortant PUUV genomes could emerge in the bank voles that inhabit the forests on the watershed between the Kazanka River and Myosha River. These findings could facilitate the search for the naturally occurring reassortants of PUUV in bank vole populations.

## 1. Introduction

Hemorrhagic fever with renal syndrome (HFRS) remains a prevalent zoonosis in the Russian Federation (RF), where the cases are registered in 68 out of 85 regions [1]. During 1997–2013, a total of 13,930 cases of HFRS were diagnosed in the Republic of Tatarstan (RT), RF [2]. HFRS counts continued to rise in 2019, when 120.8% more cases were reported compared to 2018 [3]. The HFRS infection index in the RT was 11.54 per 100 thousand people in 2020 alone [4]. The RT is ranked fourth in the registered HFRS cases after Udmurtia, Bashkiria, and Mordovia in the Volga Federal District (VFD), RF.

*Puumala orthohantavirus* (PUUV) is a member of the *Hantaviridae* family, order *Bunyavirales*, and a primary zoonotic pathogen causing HFRS in the RT and VFD [5,6,7]. PUUV is commonly isolated from bank voles, *Myodes glareolus* [8,9], which inhabit endemic regions of RT. The PUUV genome is a tri-segmented single-stranded negative-polarity RNA, where segments are defined as small (S), medium (M), and large (L). Each segment codes for a specific protein: nucleocapsid protein (NP), a precursor of the envelope glycoproteins (Gn, Gc) and RNA-dependent RNA polymerase (RdRp), respectively [10]. Eight genetic lineages of PUUV identified are circulating in bank vole populations from Europe to western Siberia [11]. Two PUUV lineages (Russian (RUS) and Finnish (FIN)) are commonly found in the bank voles captured in the European part of RF. The RUS genetic lineage of PUUV is distributed in VFD and other regions of European Russia. In contrast, the FIN genetic lineage is isolated from bank voles in Karelia and western Siberia [6,12,13,14,15,16]. Although much is learned about PUUV strains circulating in Russia, there is a need to understand better the correlation between PUUV strains isolated from HFRS patients and bank voles from the same area [17,18]. These data could help investigate HFRS outbreaks and advance our understanding of virus spreading due to bank voles’ migration.

The major aim of this study was to elucidate the relationship between PUUV strains isolated from HFRS patients and from bank voles inhabiting the same areas in the RT. Additionally, we wanted to determine whether the infection site information and a genetic variant of PUUV obtained from HFRS patients correlate with the PUUV strain isolated from the bank voles captured in the same location. Finally, we planned to identify the location of the sites where reassortant PUUV genomes could emerge.

## 2. Results

### 2.1. Molecular Analysis of PUUV Strains

PUUV RNA was detected in 88 out of 201 (43.8%) HFRS samples. The nt sequences of complete and partial S segment coding region (CDS) were obtained for 42 PUUV strains. Moreover, the nt sequences of partial M segments were collected for 24 out of these 42 PUUV strains. All PUUV sequences were deposited in the online GenBank Database. The list of PUUV S and M segment sequences, their names, and the GenBank Database (NCBI) accession numbers are summarized in Table S3. Designation of obtained PUUV sequences included virus name, city of origin, sample number, and year of collection, i.e., PUUV/Kazan/human_RT461/2019. The short names, i.e., Hu461, will be used for PUUV isolate identification in the text of this manuscript.

### 2.2. Analysis of PUUV nt Sequences Obtained from HFRS

Analysis of PUUV S segment nt sequences. Analysis of the PUUV S segment nt sequences from HFRS patients identified five groups: A, B, C, D, and E. Groups A and C includes two subgroups each: A1 and A2, and C1 and C2, respectively (Table 1). The criteria for grouping the PUUV strains identified in patients with HFRS were, first of all, the highest values of nucleotide sequences identity for both S and M segments genome between HFRS patient strains and when compared with the strains previously identified in the natural host were grouped together. When nucleotide sequences identity among a number of strains investigated was above 96%, these strains formed a group. For the S segment nt sequence’s identity in each group was between 96.2% and 100.0% (Table S4). The identity of sequences between groups was lower, ranging from 90.6% to 98.1% (Table S4). We also found that the nt sequence identity between PUUV strains from HFRS patients and bank voles from the RT, Bashkiria and Samara oblast (Table S2) belonging to the RUS genetic lineage [6,13] was between 92.8% and 96.6%. Thus, it can be concluded that all PUUV strains identified in HFRS patients belong to the RUS genetic lineage.

**Figure 1:**
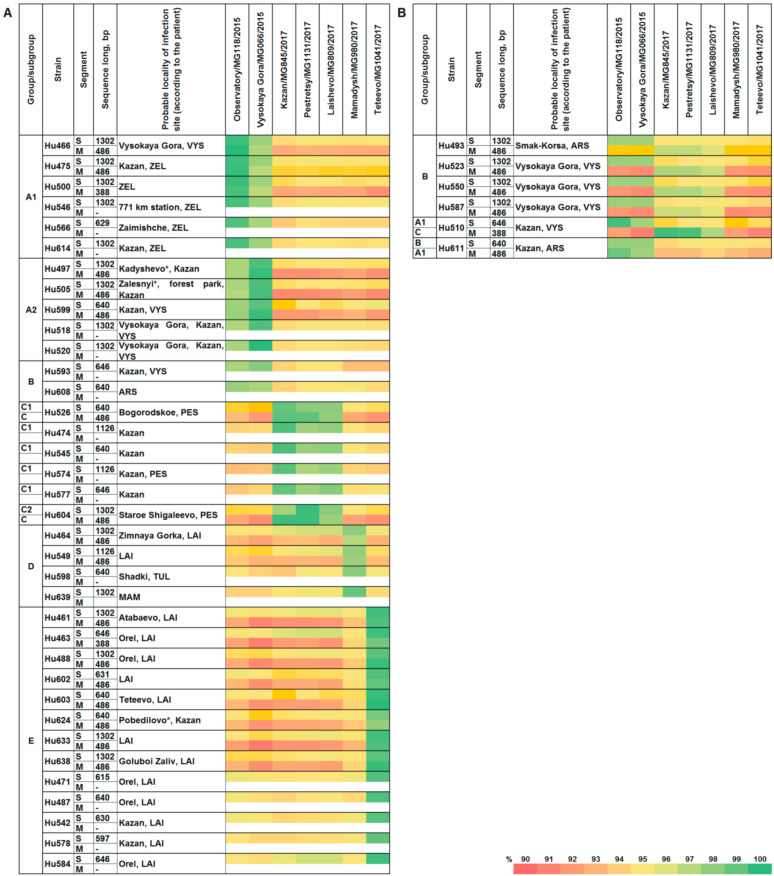
Identity of the S and M segment nucleotide sequences of the *Puumala orthohantavirus* strain obtained from hemorrhagic fever with renal syndrome patients and bank voles from several districts of the Republic of Tatarstan, %: (**A**)—original genomes; (**B**)—reassortant genomes. Districts of the RT: ARS—Arsky, LAI—Laishevsky, MAM—Mamadyshsky, PES—Pestrechinsky, TUL—Tyulachinsky, VYS—Vysokogorsky, ZEL—Zelenodolsky; *—names of Kazan city residential areas.

The nt sequence analysis of PUUV strains from HFRS cases and bank voles obtained from the several sites of the RT showed a high degree of identity (>96.7%) (Figure 1A,B).

**Figure 2:**
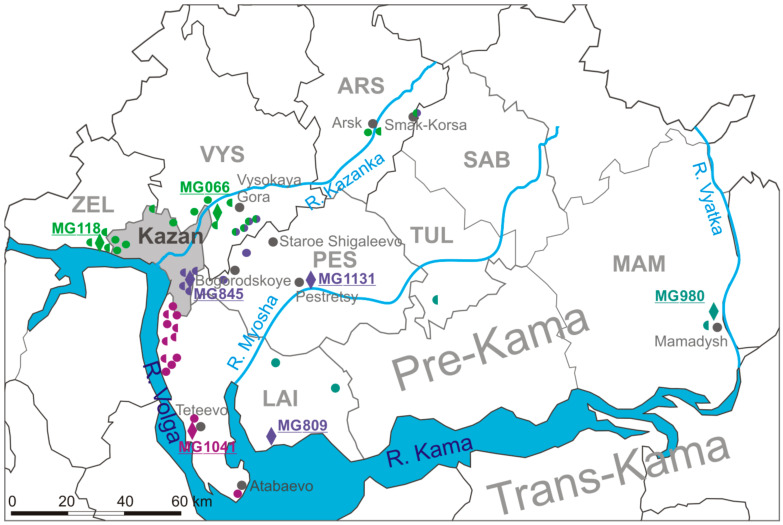
The map of *Puumala orthohantavirus* (PUUV) infection site locations and bank vole trapping sites in the Republic of Tatarstan. Semicircle—PUUV genome segment identified in hemorrhagic fever with renal syndrome patient: left semicircle—S segment; right semicircle—M segment. Multicolored circles represent reassortant genomes. Diamond—bank vole trapping site. Names starting with MG represent PUUV strains found in bank voles. Districts: ARS—Arsky, LAI—Laishevsky, MAM—Mamadyshsky, PES—Pestrechinsky, TUL—Tyulachinsky, VYS—Vysokogorsky, ZEL—Zelenodolsky.

As shown on Figure 1, PUUV strains from HFRS patients had high degree of the nt sequence identity with PUUV strains from bank voles captured in specific locations. For example, subgroup A1 strains were closely related to MG118 strain isolated from a bank vole trapped in the western outskirts of Kazan (>99.4%), while a subgroup A2 strains- to MG066 strain from the northern outskirts of Kazan (>99.1%). In group B, HFRS derived nt sequences had high identity with MG118 and MG066 (from 96.7% to 98.0%). Furthermore, high identity was found between subgroup C1 sequences and MG845 from the forest in the eastern outskirts of Kazan (>99.1%), and between subgroup C2 sequences and MG1131 from nearby Pestretsy village located 25 km east of Kazan (99.8%). HFRS case sequences in group D were similar to MG980 from Mamadysh area located 135 km east of Kazan (97.5% to 99.0%), while group E HFRS nt sequences were similar to MG1041 from nearby of Teteevo village located 30 km south of Kazan (from 98.3% to 99.7%) (Figure 2).

Analysis of the PUUV M segment nt sequences. Like the S, M segment nt sequences from HFRS formed five groups: A, B, C, D, and E. Only group A was divided into subgroups A1 and A2 (Table 1 and Table S5). We found that the identity between PUUV M segment nt sequences within groups was high (>96.6%), while it was lower between majority of these groups (from 90.7 to 93.2 %). Nt sequence identity between groups B and C was higher than the other groups (from 95.7 to 97.1%) (Table S5). 

Like the S, PUUV M segment sequences obtained from HFRS patients had higher similarity with strains from bank voles captured in several sites of the RT. For instance, the identity of nt sequences between subgroup A1 and MG118 strain was 98.6–100.0%. Sequence identity between subgroup A2 and MG066 was also high, 99.6–99.8%. In addition, nt sequences within groups B and C, when compared with MG845 and MG1131, had an identity, ranging from 99.0 to 99.8% and from 96.1 to 96.9%, respectively (Figure 1). Sequence identity of 97.5–100.0%, was found between group E and MG1041 strain. Lower identity, 96.5–97.1%, was found between sequences within group D and MG980 strain (Figure 1).

### 2.3. Analysis of the Amino Acid (aa) Sequences of PUUV from HFRS Patients and Bank Voles

PUUV N protein aa sequence. Analysis of the PUUV N protein aa sequences from HFRS cases and the bank voles (Table S2) showed more than 99.0% identity. High aa sequence identity could indicate the limited number of missense mutations in the nt sequences of the PUUV strains from HFRS and bank voles.

Interestingly, K242R aa substitution in PUUV strains detected only in groups C and E. Furthermore, we identified R242 aa residue in the PUUV strains from bank voles captured in the forest on the eastern outskirts of Kazan, the forest near Teteevo, and in the Trans-Kama area of the RT [13,14]. Another, V260I, aa substitution was discovered in the PUUV strains from C and D groups and bank vole strains MG845 and MG1131 circulating in Pestrechinsky, MG809 from Laishevsky, and MG980 found in Mamadyshsky districts of the RT [13]. The I168V aa substitution was found only in PUUV sequences from group B and in the bank vole (MG845 [13] captured in the forest in the eastern outskirt of Kazan (Figure 2). It appears that I168 aa residue is specific to PUUV found in bank voles that inhabit in this forest. Thus, this aa substitution could be used as a marker to identify the area of HFRS infection.

PUUV glycoprotein precursor (Gn/Gc) aa sequence. The Gn/Gc aa sequence identity of PUUV within each group and corresponding strains from bank voles was more than 99.0%. In contrast, aa sequences in group C had lower identity (98.4–99.4%) within the group and when compared to bank vole strains.

We also identified A1769G point mutation in Gn/Gc nt sequences of HFRS derived PUUV from groups B and C. This nt substitution led to codon change from ATT to GTT, resulting in the I577V aa substitution. I577V was also found in MG845, MG1131, and MG809 strains from bank voles in the Pre-Kama area [13]. Additionally, our analysis revealed mutation in 1973–1975 nt position leading to V645A/I aa substitution. Previous analysis showed twelve out of eighteen PUUV strains from bank voles having GTG codon at position 1973–1975 nt, which codes for Valine [13]. Interestingly, we found this codon in 14 out of 24 strains derived from HFRS. In group A, the codon was only found in the Hu611 strain, while it was present in all strains from groups C, D, and E.

In contrast, group B strains have GTA codon at 1973–1975 nt position, which, like GTG, is coding for Valine. A different codon, GCG, was found in Hu466 and Hu475 strains from subgroup A1 and MG118 bank vole strain at 1973–1975 nt position. Moreover, the codon GCC was found in Hu500 in the same position. These two codons code for Alanine. Therefore, it appears that Hu466, Hu475, Hu500, and MG118H strains have V645A aa substitution. 

Additionally, the ATA codon coding for Isoleucine was found in subgroup A2 (Hu497, Hu505, and Hu599 strains) at the same 1973–1975 nt position. Therefore, it may be suggested that the codon coding for Gn/Gc 645 aa residue has high variability. Detection of Alanine aa residue at position 645 could serve as a potential marker for mapping the site of HFRS infection.

### 2.4. Phylogenetic Analysis of the S and M Segment nt Sequences of PUUV Strains

**Figure 3:**
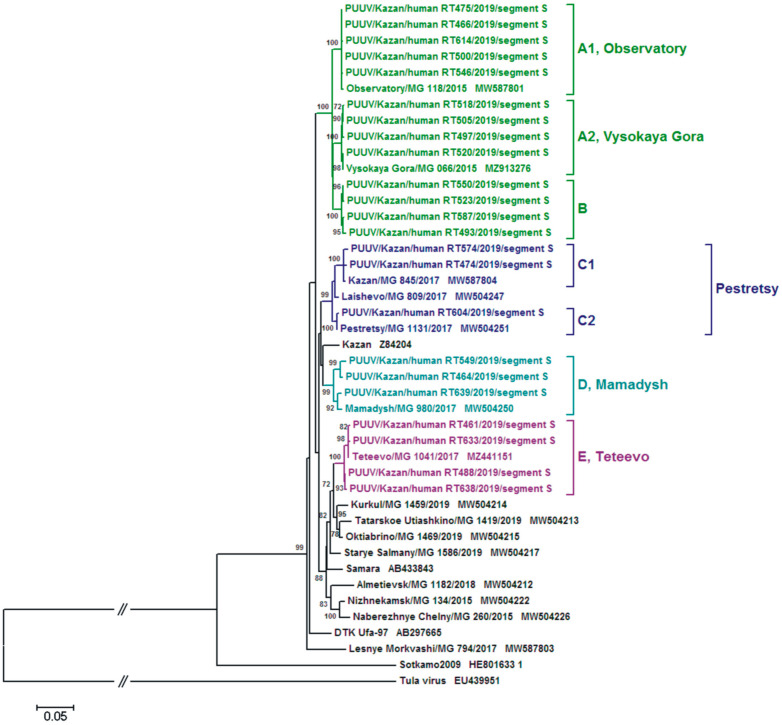
The phylogenetic tree based on the S segment CDS nt of *Puumala orthohantavirus* strains from hemorrhagic fever with renal syndrome patients and bank voles. The percentage of trees in which the associated taxa clustered together is shown next to the branches. For clarity, only taxa percentages above 70 are shown on the tree. Initial tree(s) for the heuristic search were obtained by applying the Neighbor-Joining method to a matrix of pairwise distances estimated using the Maximum Composite Likelihood (MCL) approach with 1000 bootstrap replicates. A discrete Gamma distribution was used to model evolutionary rate differences among sites (five categories (+*G*, parameter = 0.5786)). The rate variation model allowed for some sites to be evolutionarily invariable ([+*I*], 49.8447% sites). The tree is drawn to scale, with branch lengths measured in the number of substitutions per site.

**Figure 4:**
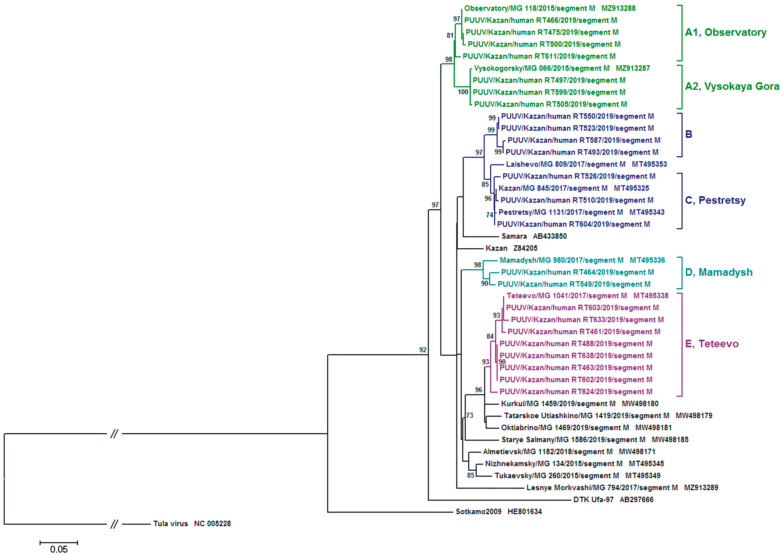
The phylogenetic tree based on the M segment partial nt sequences of *Puumala orthohantavirus* from hemorrhagic fever with renal syndrome patients and bank voles. The percentage of trees in which the associated taxa clustered together is shown next to the branches. For clarity, only taxa percentages above 70 are shown on the tree. Initial tree(s) for the heuristic search were obtained by applying the Neighbor-Joining method to a matrix of pairwise distances estimated using the Maximum Composite Likelihood (MCL) approach with 1000 bootstrap replicates. A discrete Gamma distribution was used to model evolutionary rate differences among sites (five categories (+*G*, parameter = 0.7671)). The rate variation model allowed for some sites to be evolutionarily invariable ([+*I*], 39.8943% sites). The tree is drawn to scale, with branch lengths measured in the number of substitutions per site.

The phylogenetic trees based on the PUUV S segment complete CDS (1302 nt) and the partial M segment (486 nt) sequences demonstrated moderately different topologies (Figure 3 and Figure 4, respectively).

Subgroup A1 strains formed the subclade “Observatory” together with the MG118 strain from the Observatory area on both S and M phylogenetic trees (Figure 3 and Figure 4). A subclade “Vysokaya Gora” was formed by strains from subgroups A2 and MG066. Subclades “Observatory” and “Vysokaya Gora” were located on the same branch. The S segment from subgroups C1 and C2 strains formed the subclade “Pestretsy” together with PUUV strains isolated from bank voles captured in the forest in the eastern outskirts of Kazan, Pestrechinsky and Laishevsky districts of the RT (Figure 3). Furthermore, the M segment from group C is in the same subclade as the S segment (Figure 4). Group D strains and MG980 from the Mamadysh district made the “Mamadysh” subclade (Figure 3 and Figure 4). Group E strains were clustered with the MG1041 from bank voles captured in the forest near Teteevo village and indicated as the “Teteevo” subclade on S and M segment trees (Figure 4). The positioning of “Observatory”, “Vysokaya Gora”, “Pestretsy”, and “Teteevo” subclades on both S and M segment trees was similar, while group B strains formed a distinct subclade “B” on both trees (Figure 3 and Figure 4).

It could be suggested that the variations in the tree topology are to some extent linked to the position of the subclade “Mamadysh”. On the S segment tree, the “Mamadysh” subclade is located on the branch adjacent to the “Pestretsy” subclade (Figure 3). In contrast, on the M segment tree, it forms a separate cluster closer to the “Teteevo” subclade and strains from the bank voles in the Trans-Kama area (Figure 4). Earlier, we suggested that the differences in the S and M segment trees’ topology could be the result of several PUUV strain clusters formed in the Pre-Kama area due to multidirectional primary and secondary migrations of bank voles [13]. 

Interestingly, the “B” subclade (Hu493, Hu523, Hu550, and Hu587 strains) on the S segment tree is positioned as the neighboring branch with “Vysokaya Gora” (Figure 3). In contrast, on the M segment tree, the subclade “B” is positioned as the adjacent branch to the “Pestretsy” subclade (Figure 4). The different location of the strains on phylogenetic trees suggests the possible reassortant origin of these strains [19].

## 3. Discussion

In this study, we analyzed the PUUV genome sequences from HFRS patients and bank voles collected in the RT. The nt sequences of PUUV strains from patients and bank voles trapped in the same site revealed high identity, between 96.1 and 100% (Figure 1). Phylogenetic analysis showed that most of the nt sequence from HFRS closely related to sequences of the PUUV strains previously isolated from bank voles in the RT. Our results corroborate previous reports of high similarity between PUUV strain sequences from HFRS and bank voles collected during an outbreak in 2010 in Thuringia, Germany [20]. 

It should be noted that most PUUV strains from HFRS patients were clustered in groups A, B, and E. Sixteen out of 42 (38.1%) S segment sequences (groups A and B) were obtained from infection sites located in the northern part of Kazan and adjacent parts of Zelenodolsky and Vysokogorsky districts. Furthermore, 13 out of 42 (31.0%) of these sequences (group E) were identified in HFRS with infection sites located along the left bank of the Volga River south of Kazan. Forests and parks on these territories are often used as recreation areas for city residents. The recreational use of these locations could explain why most (~70%) of PUUV strains were isolated from HFRS patients living nearby.

We established that the information about infection sites obtained from the patients could be used to map the PUUV carrying bank vole habitat. Most of the PUUV strains from HFRS were strongly linked to geographical location (Figure 2). This observation suggested that the PUUV strains circulating on the southern territory of the Arsky district are closely related to strains previously found in bank voles in Zelenodolsky and Vysokogorsky districts. Similarly, PUUV strains related to MG845 and MG1131 strains appear to be circulating in bank voles near Bogorodskoe and Staroe Shigaleevo villages in the Pestrechinsky district. PUUV strains related to those obtained from the forests near Teteevo village are also circulated in bank vole populations in the forests along the left bank of the Volga River located from the southern outskirts of Kazan to Atabayevo village (Figure 2).

The habitat of bank voles carrying PUUV strains could also be mapped by using unique aa substitution as markers. We found that one of these markers could be aa mutations K242R and V260I in the N protein sequence, as well as I577V and V645A/I in the Gn/Gc. Our data support the previous observation where two PUUV sublineages of the Central European lineage, containing Q64 and R64 aa residues in the N protein, were found in two distinct areas [21]. In another study, PUUV strains of the Alpe-Adria lineage containing D238 and E238 aa residues were identified in HFRS from different areas in Austria [17]. Our data also demonstrate that these PUUV strains with unique aa substitutions are evolutionarily stable since they were identified in bank voles trapped in 2015–2018 and HFRS patients during the 2019 outbreak (Tables S2 and S3). This is consistent with the results of Castel et al., showing the existence of evolutionarily stable PUUV strains in locations of northeastern France [22]. These unique markers of locally circulating PUUV strains could be used to detect the site of HFRS infection and tested whether to have an immunological and clinical impact. In the investigation of a fatal case, amino acid substitutions such as P233A in the N protein and D9G, D50E, K59R and N79S in the non-structural protein were found in the PUUV strains identified in a patient with HFRS. Similarly, two unique amino acid substitutions were identified T75S and V248A, located in the N terminal of the Gn ectodomain [23]. However, it still remains unclear whether the mutations in the N protein and Gn/Gc protein of the PUUV genome could change the properties of the virus in nature. In the experiment carried out by Slough et al. for HTNV and DOBV, the I532K/S1094L mutations in the Gn/Gc consistently enhanced cell surface expression, by three- to four-fold compared to the WT. Furthermore, they found the same results in U2OS and 293T, HUVECs cell lines. Moreover, the replication and propagation of double mutant Gn/Gc HNTV strains were more rapidly than single mutant virus [24]. For Gc Rift Valley fever phlebovirus, it was shown that substitutions of H857A, H778A and H1087A aa led to a decreased capability of the virus to enter the cell, however, such an effect of amino acid substitutions for orthohantaviruses has not yet been revealed [25].

Interestingly, identity between nt sequences of Hu464 and Hu549 from Laishevsky and Hu598 from Tyulachinsky districts and MG980 strain from the Mamadyshsky district was lower than 98% for both S and M segments (Figure 1A and Figure 2). Thus, we could not unambiguously associate these strains with strains from bank voles in the RT. Earlier, similar data indicating lack of clear association between PUUV strains derived from HFRS and from bank voles were demonstrated in West Germany [26]. There is limited information on the exact location of the infection site for the Hu549 from the Laishevsky and the Hu639 from the Mamadyshsky districts. However, our data suggest that PUUV strains related to MG980 could be distributed in the bank voles that inhabit Laishevsky, Tyulachinsky, Rybno-Slobodsky and Mamadyshsky districts along the right bank of the Kama River and the right tributaries of Kama River between Myosha River and Vyatka River. More data on the PUUV genome variants circulating in the bank vole populations in this area are needed to confirm this hypothesis.

It is noteworthy that the S segment of the strains Hu493, Hu608, and Hu611 identified in patients from the Arsky district is closely related to the S segment from bank voles captured in Vysokogorsky and Zelenodolsky districts. Most of the bank vole trapping sites in the Vysokogorsky and Zelenodolsky districts are located along the Kazanka River valley. According to the patients’ statements, infection sites are located along the Kazanka River valley in the Arsky district. Our data support the hypothesis that the PUUV strains from bank voles along the same river valley most likely originated from a common ancestor [14]. Thus, PUUV strains related to the strains obtained from bank voles in Vysokogorsky and Zelenodolsky districts also circulate in the bank voles in the Kazanka River valley in the Arsk district. Similarly, some related PUUV strains were detected earlier in HFRS patients and bank voles within the Vichte River valley in Germany [27]. 

**Figure 5:**
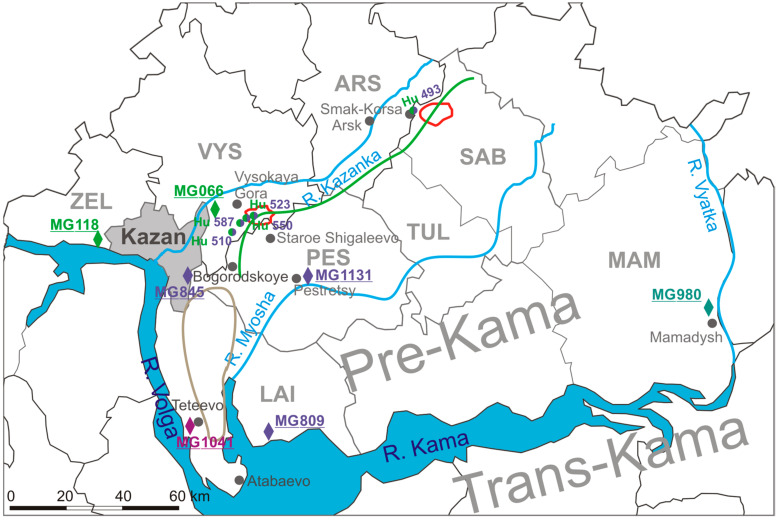
The map of forest locations on the watershed between the Kazanka River and Myosha River. The green curve line—is a watershed line between the Kazanka River and the Myosha River. Red lanes—forests located on the watershed between the Kazanka River and Myosha River. Light-brown line—an area with a small number of forests. Semicircle—*Puumala orthohantavirus* (PUUV) genome variant identified in hemorrhagic fever with renal syndrome (HFRS) patient; left semicircle—S segment; right semicircle—M segment. Diamond—PUUV strains identified in the bank vole populations. Names starting with MG represent PUUV strains found in bank voles, and names starting with Hu represent PUUV strains identified in HFRS patients. Multicolored circles and multicolored names represent reassortment genomes and PUUV strains, respectively.

Comparison of the tree topology for S and M segments suggests that the strains from group B are reassortants. Recently, PUUV reassortment was demonstrated by Razzauti et al., where the PUUV strains obtained from the bank voles were shown to be a result of genome exchange between genetic variants of the FIN lineage [28] and between strains of FIN and North Scandinavian lineages [19]. In another study, a possible reassortant PUUV strain was isolated from an HFRS patient in Tampere University Hospital, Finland [29]. Furthermore, recently, a suspected reassortant PUUV strain was identified in the Ardennes forest of northeastern France [30]. In our previous study, we described the PUUV reassortants emergence in the bank vole populations under natural conditions [14]. We predicted that the possible sites for PUUV reassortants emergence could be in the forests along the watersheds. Our data on PUUV genomes identified in HFRS patients provide more indirect evidence supporting this assumption. For example, the Hu493 strain isolated from a patient infected in the vicinity of the Smak-Korsa village possesses the S segment from group B, closely related to the S segment from the bank vole strain (MG066) captured in Kazanka River valley in Vysokogorsky district, and the M segment from group B closely related the M segment from bank vole (MG1131 and MG809) inhabiting along the Myosha River valley in Pestrechinsky and Laishevsky districts. Such a high similarity of the PUUV S and M segments with strains from different phylogenetic subclades suggests that Hu493 has a reassortment origin (Figure 1B, Figure 3 and Figure 4). It is important to note that, near Smak-Korsa village, there is only one forest located in the watershed between Kazanka and Myosha rivers (Figure 5). It is likely that a reassortant PUUV genome variant circulates in the bank voles in this forest near Smak-Korsa village.

Furthermore, Hu523, Hu550, and Hu587 from group B possess the S segment closely related to that of the MG066 strain, and the M segment closely related to that of the MG1131 strain. This could be a sign of Hu523, Hu550, and Hu587 having reassortant origin (Figure 1B, Figure 3 and Figure 4). These strains were isolated from HFRS patients infected in the Vysokaya Gora village area. Approximately 1 km east of this village, there is a forest positioned on the watershed between the Kazanka and Myosha rivers (Figure 5). There are small groves and groups of trees on both sides of the watershed. These groves encourage bank vole migrations to this forest on the watershed and make it possible to form a contact zone between different bank vole populations. We believe that these areas could serve as the sites where reassortant genomes could emerge as the result of bank voles’ infection with different PUUV strains.

An alternative mechanism considering the emergence of PUUV reassortants in HFRS patients as a result of simultaneous or consecutive infection of the humans with two virus strains seems unlikely. This mode of infection will require that three unrelated and unfamiliar individuals of different ages and occupations to visit two infection sites located several kilometers from each other. Moreover, the chance that all three patients would develop similar reassortant strains is less likely. The most plausible explanation for reassortants to appear in HFRS patients is patients’ exposure to reassortant PUUV strains that emerged in the bank voles in the contact zones.

The reassortant genome of the Hu510 strain differs from four strains discussed above. In the Hu510, the S segment is from subgroup A1 and is closely related to the S segment of the MG118 from the bank vole in the western outskirts of Kazan. In contrast, the M segment is from group C and is closely related to the M segment of the MG845 from the bank vole in the eastern outskirts of Kazan (Figure 1B and Figure 5). We suggest that the infection site is in one of the forests, squares or parks within the boundaries of Kazan city. However, the exact location of this site still needs to be determined.

Moreover, the location of the infection site for the Hu611 strain remains undetermined. According to the patient’s statement, the infection could have occurred in the Arsky district. The S segment is clustered in group B, while the M segment is in subgroup A1 (Figure 1B). Strains of groups A and B are commonly found in bank voles from the Kazanka River valley. In our previous report [14], we suggested that contact zones between bank vole populations carrying closely related PUUV strains could be formed along the river valley. It is possible that the Hu611 is also a reassortant, but its ancestral strains were closely related to each other, and the contact zone where this strain was formed could be located in the Kazanka River valley.

It is also interesting to note that there were no PUUV reassortants found between strains in groups A/B and E, as well as between groups C and E. We suggest that this could be explained by the existence of physical obstacles preventing the formation of contact zones between the corresponding bank vole populations. For example, Kazan city serves as a barrier between habitats where PUUV groups A/B and E strains are circulating in bank voles (Figure 2 and Figure 5). Furthermore, a vast territory with a small number of forests uninhabitable for the bank voles separates areas where PUUV from group C and E are circulating. This territory could be an obstacle preventing contact between infected bank voles from the populations near the Teteevo village and the Myosha River valley (Figure 5). Our data provide evidence suggesting that habitats in forested areas along the watersheds could be sites where bank voles become infected with several PUUV strains, leading to emergence of reassortants.

## 4. Materials and Methods

Samples. Blood samples were collected from 201 HFRS patients (121 male and 80 female) hospitalized at the Agafonov Republican Clinical Hospital for Infectious Diseases in Kazan city, RT, in 2019. Diagnosis of HFRS was established based on clinical presentation and by detection of anti-HFRS antibodies. All samples were obtained during the acute phase of the disease. 

Ethics statement. Sample collection was performed according to a protocol approved by the Institutional Review Board of the Kazan Federal University. Informed consent was obtained from each subject according to the guidelines approved under this protocol (Article 20, Federal Law “Protection of Health Rights of Citizens of Russian Federation” N323-FZ, 21 November 2011).

RNA Extraction, cDNA Synthesis, and RT-PCR. Total RNA was isolated from HFRS blood samples using TRIzol Reagent (Invitrogen Life Technologies™, Waltham, MA, USA) according to the manufacturer’s recommendations. cDNA was synthesized with Thermo Scientific RevertAid Reverse Transcriptase (Thermo Fisher Scientific, Waltham, MA, USA) as specified by the manufacturer. Nested PCR was performed using ScreenMix (Evrogen, Moscow, Russia), following the manufacturer’s instructions. The PCR amplicons were sequenced using ABI PRISM 310 big Dye Terminator 3.1 sequencing kit (ABI, Waltham, MA, USA). Primers used in the current study are summarized in Table S1.

Phylogenetic Analysis. PUUV nucleotide (nt) sequence alignments were conducted using the Clustal W algorithm in the MegAlign program (DNASTAR software package Lasergene (DNASTAR, Madison, WI, USA; https://www.dnastar.com/, accessed on 27 February 2022)) and in MEGA v6.0 [31]. Phylogenetic trees were generated using the Maximum Likelihood method based on the Tamura-Nei model in MEGA v6.0. [31]. Furthermore, S and M segment sequences of the GenBank PUUV strains previously obtained from bank voles in the RT and other regions of Russia were used for comparison (Table S2). Sequences of *Tula orthohantavirus* S and M segments were used as outgroups (Table S2). Detailed and abbreviated names of the reference strains are given in Table S2. For convenience, we will use abbreviated names of the strains isolated from bank voles in the RT, for example, MG118.

## 5. Conclusions

Our data demonstrate that the information about the human infection sites and the identified PUUV strains from HFRS patients could be used to determine the area where these strains could be circulating in bank voles. Moreover, our data confirmed that unique aa mutations could be used to map the sites of HFRS infection. The reassortant PUUV genomes found in HFRS patients have yet to be detected in the bank voles in the RT. However, we have obtained indirect evidence supporting our previous assumption that the reassortant PUUV genomes could emerge in contact zones located in the forests on the watersheds. In particular, the location of the reassortant PUUV infection site suggests that these genomes could emerge in the bank voles inhabiting forests on the watershed between the Kazanka River and Myosha River. These findings could facilitate the search for the naturally occurring reassortants of PUUV in bank vole populations.

## Figures and Tables

**Table 1:** Hemorrhagic fever with renal syndrome (HFRS) derived *Puumala orthohantavirus* (PUUV) strain groups based on nt sequence identity.

Group	Subgroup	Segment	PUUV Strain
A	A1	S, complete S, partial S, partial	Hu466, Hu475, Hu500, Hu546, Hu614 Hu566 Hu510
	A2	S, complete S, partial	Hu497, Hu505, Hu518, Hu520 Hu599
B		S, complete S, partial S, partial	Hu493, Hu523, Hu550, Hu587 Hu608, Hu611 Hu593
C	C1	S, complete S, partial S, partial	Hu474, Hu574 Hu526, Hu545 Hu577
	C2	S, complete	Hu604
D		S, complete S, partial	Hu464, Hu549, Hu639 Hu598
E		S, complete S, partial S, partial	Hu461, Hu488, Hu633, Hu638 Hu471, Hu487, Hu542, Hu578, Hu602, Hu603, Hu624 Hu463, Hu584
A	A1	M, partial	Hu466, Hu475, Hu500, Hu611
	A2	M, partial	Hu497, Hu505, Hu599
B		M, partial	Hu493, Hu523, Hu550, Hu587
C		M, partial	Hu510, Hu526, Hu604
D		M, partial	Hu464, Hu549
E		M, partial	Hu461, Hu463, Hu488, Hu602, Hu603, Hu624, Hu633, Hu638

## Data Availability

The original contributions presented in the study are included in the article/Supplementary Materials and available in NCBI database, Further inquiries can be directed to the corresponding author.

## Supplementary Materials

The supporting information can be downloaded at: https://www.mdpi.com/article/10.3390/ijms24021018/s1. Reference [32] is cited in the Supplementary Materials.
