# Supplementary material for: Puumala Orthohantavirus Reassortant Genome Variants Likely Emerging in the Watershed Forests
Source: Int J Mol Sci. 2023 Jan 5;24(2):1018. doi: 10.3390/ijms24021018 (PMC9865142; doi:10.3390/ijms24021018)
Supplement: Supplementary file 1 [file ijms-24-01018-s001.zip › ijms-2063649-supplementary.pdf]

Supplement

Table S1. Primers used for the RT-PCR amplification and sequencing

Primer designation	Nucleotide sequence, 5′ → 3′	Location [a]	Reference
Forward			
PuuV-S-F1	tagtagtagactccttgaaaagc	1-23	[1]
PuuV-S-F41	agctactacgagaacaactgg	21-41	[1]
PuuV-For	ctgcaagccaggcaacaaacagtgtcagca	172-201	[2]
4S-F3	gcaatggaggataaactcgc	199-218	[1]
39S-F3	ggccaaaacatctatatgtatcc	560-582	This paper
PUUV-S-F704	aacatcatgagtccagtaatggg	682-704	[1]
PUUV-S-F763	cagagagaattagggagtttatgg	740-763	This paper
PUUV-S-F991	ggtatttgcctgtgcaccag	972-991	This paper
69S-F3	ttatggcatctaaaactgtgg	1079-1099	[1]
Reverse			
PUUV-S-R228	acagcatctgccattcttctc	228-248	This paper
4S-B3	gtgaaccctattacatagactcc	409-431	This paper
32S-B3	attgatatcctcaaatgatgtgt	527-549	This paper
5S-B3	ggccagtctttaagcaagaaag	719-740	[1]
PuuV-Rev	gtctgccacatgatttttgtcaagcacatc	865-894	[2]
PuuV-S-R972	tctggtgcacaggcaaatacc	972-992	This paper
69S-B3	gatatctcttttaccttctggtc	1297-1319	This paper
PuuV-S-R1496	gtataattccagttaacccctg	1496-1517	[1]
M segment			
Forward			
F1452-PuuV-M	tctttaatcccaggagttgc	1351-1470	[3]
1M-F3	ggtatatgagcctatgcaagg	1888-1907	This paper
F2143-PuuM	acagaatccagctaatgaacaag	2143-2165	This paper

Reverse			
1M-B3	gctgtctcctatatgtatagct	2120-2141	This paper
PUUV-M-R2359	caatcaggtgggttacadccc	2359-2379	This paper
R2582 PUUV-M	aaattgtccctattaaacacac	2561-2582	[3]

[a] Numbering corresponds to the position of nucleotide (nt) sequences on the PUUV strain Puu/Kazan used for comparison (Genbank Accession No Z84204 and Z84205 for S and M segment, respectively).

Table S2. List of the *Puumala orthohantavirus* (PUUV) sequences used for phylogenetic analysis as references

Strain sequence	Short name	GenBank Accession No
PUUV/Observatory/MG_118/2015/segment S	MG118	MW587801
PUUV/Nizhnekamsk/MG_134/2015/segment S	MG134	MW504222
PUUV/Naberezhnye Chelny/MG_260/2015/segment S	MG260	MW504226
PUUV/Lesnye Morkvashi/MG_794/2017/segment S	MG794	MW587803
PUUV/Laishevo/MG_809/2017/segment S	MG809	MW504247
PUUV/Kazan/MG_845/2017/segment S	MG845	MW587804
PUUV/Mamadysh/MG_980/2017/segment S	MG980	MW504250
PUUV/Pestretsy/MG_1131/2017/segment S	MG1131	MW504251
PUUV/Almetievsk/MG_1182/2018/segment S	MG1182	MW504212
PUUV/Teteevo/MG_1041/2017/segment S	MG1152	MZ441151
PUUV/Tatarskoe Utiashkino/MG_1419/2019/segment S	MG1419	MW504213
PUUV/Kurkul/MG_1459/2019/segment S	MG1459	MW504214
PUUV/Oktiabrino/MG_1469/2019/segment S	MG1469	MW504215
PUUV/Starye Salmany/MG_1586/2019/segment S	MG1586	MW504217
PUUV/Vysokaya Gora/MG_066/2015/segment S	MG066	MZ913276
Puu/Kazan	Kazan	Z84204
Samara_49/CG/2005	Samara	AB433843

DTK/Ufa-97	Ufa	AB297665
Sotkamo 2009	Sotkamo	HE801633
Tula virus	Tula	EU439951
PUUV/Vysokogorsky/MG_066/2015/segment M	MG066	MZ913287
PUUV/Observatory/MG_118/2015/segment M	MG118	MZ913288
PUUV/Nizhnekamsky/MG_134/2015/segment M	MG134	MT495345
PUUV/Tukaevsky/MG_260/2015/segment M	MG260	MT495349
Lesnye Morkvashi/MG_794/2017/segment M	MG794	MZ913289
PUUV/Laishevo/MG_809/2017/segment M	MG809	MT495353
PUUV/Kazan/MG_845/2017/segment M	MG845	MT495325
PUUV/Mamadysh/MG_980/2017/segment M	MG980	MT495336
PUUV/Pestretsy/MG_1131/2017/segment M	MG1131	MT495343
PUUV/Almetievsk/MG_1182/2018/segment M	MG1182	MW498171
PUUV/Teteevo/MG_1041/2017/segment M	MG1152	MT495338
PUUV/Tatarskoe Utiashkino/MG_1419/2019/segment M	MG1419	MW498179
PUUV/Kurkul/MG_1459/2019/segment M	MG1459	MW498180
PUUV/Oktiabrino/MG_1469/2019/segment M	MG1469	MW498181
PUUV/Starye Salmany/MG_1586/2019/segment M	MG1586	MW498185
Puu/Kazan	Kazan	Z84205
Samara_49/CG/2005	Samara	AB433850
DTK/Ufa-97	Ufa	AB297666
Sotkamo 2009	Sotkamo	HE801634
Tula virus	Tula	NC_005228

Table S3. List of the obtained PUUV strains and GenBank accession No of S and M segment sequences.

Strain	Short	Segment	Accession No

	name		
S segment			
PUUV/Kazan/human_RT461/2019	Hu461	S, complete CDS	MW587793
PUUV/Kazan/human_RT464/2019	Hu464	S, complete CDS	MZ913278
PUUV/Kazan/human_RT466/2019	Hu466	S, complete CDS	MZ913279
PUUV/Kazan/human_RT475/2019	Hu475	S, complete CDS	MZ913280
PUUV/Kazan/human_RT488/2019	Hu488	S, complete CDS	MW587794
PUUV/Kazan/human_RT493/2019	Hu493	S, complete CDS	MW587795
PUUV/Kazan/human_RT497/2019	Hu497	S, complete CDS	MW587796
PUUV/Kazan/human_RT505/2019	Hu505	S, complete CDS	MW587797
PUUV/Kazan/human_RT523/2019	Hu523	S, complete CDS	MZ913281
PUUV/Kazan/human_RT550/2019	Hu550	S, complete CDS	MZ913282
PUUV/Kazan/human_RT604/2019	Hu604	S, complete CDS	MZ913285
PUUV/Kazan/human_RT638/2019	Hu638	S, complete CDS	MZ913286
PUUV/Kazan/human_RT587/2019	Hu587	S, partial CDS	OP459283
PUUV/Kazan/human_RT633/2019	Hu633	S, partial CDS	OP459293
PUUV/Kazan/human_RT463/2019	Hu463	S, partial CDS	OP459273
PUUV/Kazan/human_RT471/2019	Hu471	S, partial CDS	OP459274
PUUV/Kazan/human_RT474/2019	Hu474	S, partial CDS	MW587807
PUUV/Kazan/human_RT487/2019	Hu487	S, partial CDS	OP459275
PUUV/Kazan/human_RT500/2019	Hu500	S, complete CDS	OP459276
PUUV/Kazan/human_RT510/2019	Hu510	S, partial CDS	OP459277
PUUV/Kazan/human_RT518/2019	Hu518	S, complete CDS	MW587798
PUUV/Kazan/human_RT520/2019	Hu520	S, complete CDS	MW587799
PUUV/Kazan/human_RT526/2019	Hu526	S, partial CDS	MW587809
PUUV/Kazan/human_RT542/2019	Hu542	S, partial CDS	OP459295
PUUV/Kazan/human_RT545/2019	Hu545	S, partial CDS	MW587810
PUUV/Kazan/human_RT546/2019	Hu546	S, complete CDS	MW587800
PUUV/Kazan/human_RT549/2019	Hu549	S, partial CDS	OP459278

PUUV/Kazan/human_RT566/2019	Hu566	S, partial CDS	OP459279
PUUV/Kazan/human_RT574/2019	Hu574	S, partial CDS	MW587800
PUUV/Kazan/human_RT577/2019	Hu577	S, partial CDS	OP459280
PUUV/Kazan/human_RT578/2019	Hu578	S, partial CDS	OP459281
PUUV/Kazan/human_RT584/2019	Hu584	S, partial CDS	OP459282
PUUV/Kazan/human_RT593/2019	Hu593	S, partial CDS	OP459284
PUUV/Kazan/human_RT598/2019	Hu598	S, partial CDS	OP459285
PUUV/Kazan/human_RT599/2019	Hu599	S, partial CDS	OP459286
PUUV/Kazan/human_RT602/2019	Hu602	S, partial CDS	OP459287
PUUV/Kazan/human_RT603/2019	Hu603	S, partial CDS	OP459288
PUUV/Kazan/human_RT608/2019	Hu608	S, partial CDS	OP459289
PUUV/Kazan/human_RT611/2019	Hu611	S, partial CDS	OP459290
PUUV/Kazan/human_RT614/2019	Hu614	S, complete CDS	OP459291
PUUV/Kazan/human_RT624/2019	Hu624	S, partial CDS	OP459292
PUUV/Kazan/human_RT639/2019	Hu639	S, complete CDS	OP459294
**M segment**			
PUUV/Kazan/human_RT461/2019	Hu461	M, partial CDS	MZ913292
PUUV/Kazan/human_RT464/2019	Hu464	M, partial CDS	MZ913293
PUUV/Kazan/human_RT466/2019	Hu466	M, partial CDS	MZ913294
PUUV/Kazan/human_RT475/2019	Hu475	M, partial CDS	MZ913295
PUUV/Kazan/human_RT488/2019	Hu488	M, partial CDS	MZ913296
PUUV/Kazan/human_RT493/2019	Hu493	M, partial CDS	MZ913297
PUUV/Kazan/human_RT497/2019	Hu497	M, partial CDS	MZ913298
PUUV/Kazan/human_RT505/2019	Hu505	M, partial CDS	MZ913299
PUUV/Kazan/human_RT523/2019	Hu523	M, partial CDS	MZ913300
PUUV/Kazan/human_RT550/2019	Hu550	M, partial CDS	MZ913301
PUUV/Kazan/human_RT604/2019	Hu604	M, partial CDS	MZ913304
PUUV/Kazan/human_RT638/2019	Hu638	M, partial CDS	MZ913305
PUUV/Kazan/human_RT463/2019	Hu463	M, partial CDS	OP441075

PUUV/Kazan/human_RT500/2019	Hu500	M, partial CDS	OP441076
PUUV/Kazan/human_RT510/2019	Hu510	M, partial CDS	OP484977
PUUV/Kazan/human_RT526/2019	Hu526	M, partial CDS	OP441077
PUUV/Kazan/human_RT549/2019	Hu549	M, partial CDS	OP441078
PUUV/Kazan/human_RT587/2019	Hu587	M, partial CDS	OP441079
PUUV/Kazan/human_RT599/2019	Hu599	M, partial CDS	OP441080
PUUV/Kazan/human_RT602/2019	Hu602	M, partial CDS	OP441081
PUUV/Kazan/human_RT603/2019	Hu603	M, partial CDS	OP441082
PUUV/Kazan/human_RT611/2019	Hu611	M, partial CDS	OP484974
PUUV/Kazan/human_RT624/2019	Hu624	M, partial CDS	OP484975
PUUV/Kazan/human_RT633/2019	Hu633	M, partial CDS	OP484976

Table S4. Values of the identity of the S segment nt sequence between groups of PUUV strains identified in hemorrhagic fever with renal syndrome (HFRS) patients, %

Group/ subgroup	A1	A2	B	C1	C2	D	E
A1	99.4-100.0	97.0-98.1	95.3-98.1	92.5-94.7	94.3-94.9	92.5-94.6	93.8-95.3
A2		99.1-99.9	95.6-98.1	91.9-94.7	93.9-95.0	93.4-94.5	93.4-95.0
B			97.7-99.9	91.9-94.4	94.1-95.3	90.6-94.7	91.2-95.0
C1				98.4-100.0	97.4-97.8	91.9-95.1	93.4-97.5
C2					100.0	94.2-95.7	93.6-96.1
D						97.9-99.0	93.1-95.3
E							96.2-100.0

Table S5. Values of the identity of the M segment nt sequence between groups of PUUV strains identified in HFRS patients, %

Group	A1	A2	B	C	D	E
A1	97.9-99.7	96.6-97.3	91.0-92.0	92.0-93.2	92.5-93.2	91.2-92.6
A2		99.8-100.0	91.2-91.6	90.7-91.8	92.0-92.4	90.5-91.6
B			98.8-100.0	95.7-97.1	91.4-92.2	91.0-91.8
C				98.7-99.5	92.0-93.2	90.7-92.2
D					98.1	92.0-93.0
E						96.9-100.0

Reference

1. Davidyuk, Y.; Shamsutdinov, A.; Kabwe, E.; Ismagilova, R.; Martynova, E.; Belyaev, A.; Shuralev, E.; Trifonov, V.; Savitskaya, T.; Isaeva, G., et al. Prevalence of the Puumala orthohantavirus Strains in the Pre-Kama Area of the Republic of Tatarstan, Russia. *Pathogens* **2020**, *9*, doi:10.3390/pathogens9070540.
2. Kariwa, H.; Tkachenko, E.A.; Morozov, V.G.; Seto, T.; Tanikawa, Y.; Kolominov, S.I.; Belov, S.N.; Nakamura, I.; Hashimoto, N.; Balakiev, A.E., et al. Epidemiological study of hantavirus infection in the Samara Region of European Russia. *The Journal of veterinary medical science* **2009**, *71*, 1569-1578, doi:10.1292/jvms.001569.
3. Kabwe, E., Davidyuk, Y.N. and Morzunov, S.P. Genome variations of Puumala virus strains circulating in Nizhnekamsky and Tukaevsky districts of the Republic of Tatarstan. *Uchenye Zapiski Kazanskogo Universiteta. Seriya Estestvennye Nauki* **2018**, *160*, 373-385.
